# Toxocariasis: America's Most Common Neglected Infection of Poverty and a Helminthiasis of Global Importance?

**DOI:** 10.1371/journal.pntd.0000400

**Published:** 2009-03-31

**Authors:** Peter J. Hotez, Patricia P. Wilkins

**Affiliations:** 1 Department of Microbiology, Immunology, and Tropical Medicine, The George Washington University, Washington, D.C., United States of America; 2 Sabin Vaccine Institute, Washington, D.C., United States of America; 3 Division of Parasitic Diseases, National Center for Zoonotic, Vector-Borne and Enteric Diseases, Centers for Disease Control and Prevention, Atlanta, Georgia, United States of America

New information indicates that toxocariasis is the most common human parasitic worm infection in the United States, affecting millions of Americans living in poverty. The infection is also highly prevalent in many developing countries and its global importance may be greatly underestimated.

**Figure 1:**
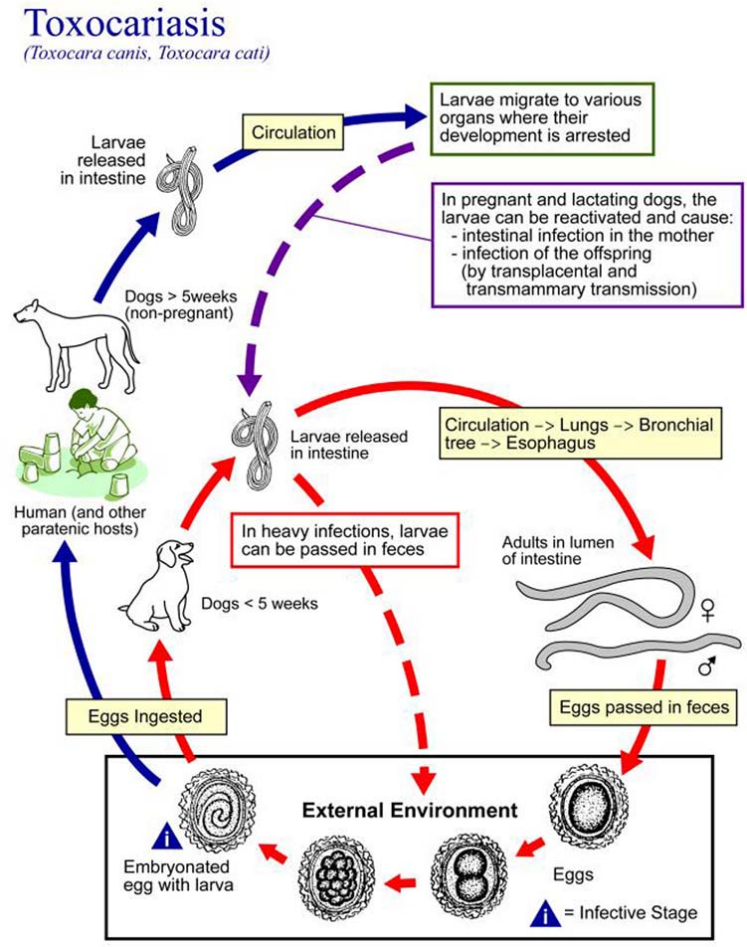
The Life Cycle of Human Infection with *Toxocara canis*. From the Public Health Image Library of the CDC, http://phil.cdc.gov.

**Figure 2:**
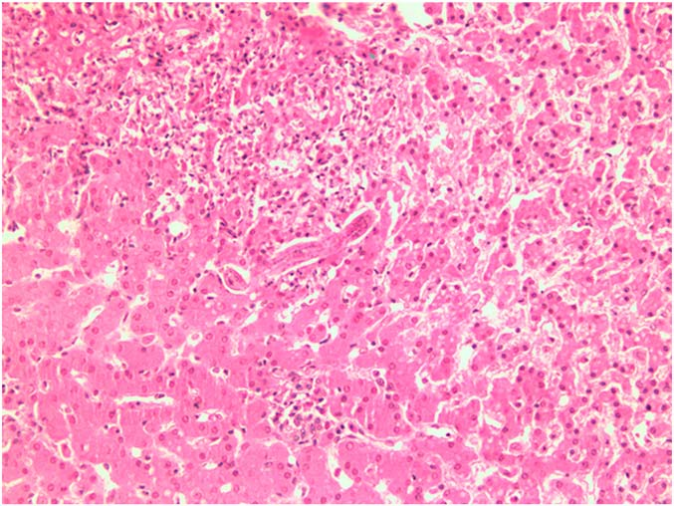
Toxocara Larva in Liver of Child Necropsied in New Zealand. Larva discovered at some distance from lesion. Image courtesy of CDC and DPDx.

Toxocariasis results from zoonotic transmission of the roundworms, *Toxocara canis* and *T. cati* from dogs and cats, respectively. Infection occurs when humans accidentally ingest the microscopic, oval and thick-shelled-embryonated eggs (shed in dog and cat feces) containing *Toxocara* larvae by hand-to-mouth contact. Children are particularly prone to infection because they are exposed to the eggs on sandboxes and playgrounds contaminated with dog and cat feces [1],[2]. After ingestion of the eggs, the released larvae penetrate the intestine and migrate through the liver, lungs, and central nervous system (Figure 1). The resulting host inflammatory response ultimately overwhelms and either kills the migrating larvae or forces them into a state of arrested development, but not before they cause both mechanical and immunopathological damage to the issues (Figure 2).

There are two “classical” clinical syndromes resulting from infection [1],[2]. Visceral larva migrans occurs most commonly in young children and results in hepatitis and pneumonitis as the larvae migrate through the liver and lungs, respectively. The full clinical presentation of toxocariasis includes hepatomegaly and pulmonary infiltrates or nodules accompanied by cough, wheezing, eosinophilia, lymphadenopathy, and fever. Larval entry into the central nervous system can also result in meningoencephalitis and cerebritis manifesting as seizures [3],[4]. Ocular larva migrans occurs more frequently in older children and adolescents and may result from the migration of even a single larva in the eye. The resulting inflammation presents clinically as either a granuloma or a granulomatous larval track in the retina or as a condition of the vitreous that resembles endophthalmitis [5],[6]. Neither visceral larva migrans nor ocular larva migrans are considered common conditions, although the incidence of the former has not been determined and it has been estimated at just under 1 per 10,000 annually for the ocular form [6]. Far more common is non-classic, or covert toxocariasis, which may manifest with only some of the clinical features found in visceral larva migrans, especially wheezing, pulmonary infiltrates, and eosinophilia [2]. Because these features are also the hallmark of childhood asthma, some investigators have hypothesized or in some cases have actually shown a link with *Toxocara* infection [2], [7]–[14]. Similarly, some of the central nervous system features of toxocariasis have been implicated as a cause of occult seizures, mental retardation, and developmental delays [3],[4],[15]. Because pica is a risk factor for both toxocariasis and lead ingestion [16], it is possible that an element of the cognitive and mental deficits ascribed to toxocariasis may partially result from plumbism.

There are an estimated 73 million dogs and 90 million cats in the United States [17]. Many pups are born with congenital canine toxocariasis and large numbers of both dogs and cats are either stray animals or pets that are not routinely dewormed as recommended by the American Veterinary Medical Association [18]. Such huge numbers of *Toxocara*-infected dogs and cats serve as rich sources of eggs in the environment, which have been recovered in poor urban areas [16] as well as in rural areas, especially in the American South and Appalachia [19]–[21]. Most of the prevalence estimates for toxocariasis in the US are based on serological surveys with banked sera that detect *Toxocara*-specific antibodies [17],[20],[22]. The enzyme immunoassay (EIA) using *T. canis* excretory-secretory (TES) antigens from infective-stage larvae is the most useful diagnostic test for toxocaral visceral larva migrans and ocular larva migrans and is the assay used by most commercial reference laboratories in the US, including the reference laboratory at the US Centers for Disease Control and Prevention (CDC) [17], [20], [22]–[31]. Results from the CDC EIA measure total immunoglobulin antibodies and are reported as a titer; the assay detects infections caused by both *T. canis* and *T. cati*. For visceral larva migrans and some forms of covert toxocariasis, the sensitivity and specificity of the *Toxocara* EIA is estimated at 78% and 92%, respectively, at a titer of 1∶32 [17],[22],[26],[27]. The sensitivity of the EIA for ocular larva migrans, however, is considerably less [1],[28]. Following initial infection, *Toxocara* larvae migrate through host tissues for several months, and ultimately generate a host granulomatous response, which blocks further larval migration. However, the larvae may remain alive within the host for months, and host antibody levels may remain strongly positive for 2 or 3 years or more [17],[31]. Therefore, in the CDC EIA, the presence of antibody titers greater than 1∶32 may be considered reflective of active infection, although we are not aware of careful studies that have determined the length of persistent toxocaral antibodies over long periods of time.

Using a nationally representative set of banked sera, the CDC has undertaken two major national surveys for toxocariasis [17],[20],[22]. The first was reported more than 20 years ago using sera from children aged 1 to 11 that were collected during the first Health and Nutrition Examination Survey (HANES I) of over 23,000 persons 1 to 74 years of age in 35 geographic regions from 1971 to 1973 [20]. Nationwide, the overall prevalence was found to vary between 4.6% and 7.3%, but ranged as high as 10% in the American South and over 30% for socioeconomically disadvantaged African American children [20]. Higher seroprevalence was also linked to markers of low socioeconomic status, including poverty and crowding and lower educational level for head of household [20]. In 2008, the CDC again reported on *Toxocara* seroprevalence from the Third National Health and Nutrition Examination Survey (NHANES III), a cross-sectional survey conducted between 1988 and 1994 [17],[22]. The survey sampled at higher rates specific minority groups (e.g., non-Hispanic blacks and Mexican Americans) and age groups (young children and the elderly) [17]. Based on a representative sample of just over 20,000 in individuals over the age of 6, the overall seroprevalence was 13.9% [17],[22], suggesting that tens of millions of Americans are infected with *Toxocara*. However, the seroprevalence was found to be considerably higher among non-Hispanic blacks and people living in poverty. Based on the number of African Americans living in poverty in the US, we calculated that as many as 2.8 million have toxocariasis, making this disease one of the most common infections among any underrepresented minority groups [32]. In a separate study conducted in the 1990s, high rates of toxocariasis were also found among inner city Hispanic populations in Bridgeport and New Haven, Connecticut, especially among Puerto Rican immigrants [14]. High rates of the infection were noted previously to occur in Puerto Rico [33]. Given its proposed links with asthma and developmental delays, human toxocariasis may represent a health disparity of staggering proportions, possibly associated with the high frequency of asthma and developmental delays noted among African Americans and some Hispanic groups living in poverty [34]–[37]. The earlier association noted between toxocariasis and elevated lead levels observed in the HANES I study was confirmed in the NHANES III serum bank data, as was an interesting association between toxocariasis and co-infection with toxoplasmosis [17],[22]. The health and developmental impact of these co-factors also warrants further investigation. Globally, high rates of toxocariasis has been noted in middle-income countries, with prevalence rates reaching 40% or higher in Indonesia and Brazil [30],[38]. Although there are few reported studies from low-income countries, it is of great interest to determine whether infection rates with *Toxocara* may exceed some of the better known human soil-transmitted helminth infections such as ascariasis, trichuriasis, or hookworm infection.

While the NHANES studies indicate that toxocariasis continues to persist and is under-recognized as a health problem, a full appreciation of the US and global burden of disease caused by toxocariasis demands improved serodiagnostic tools. In the US, EIA testing is not widely available because of the limited capacity for parasitic disease diagnosis in the US and the limited availability of antigen made from *T. canis* larvae. In addition, the existing assays have a low sensitivity for detecting ocular larva migrans, so some true cases remain undiagnosed and the approximations of national seroprevalence are underestimated. These features, together with the observation that many physicians in the US are not knowledgeable about the infection, helps to preserve the neglected status of toxocariasis. In developing countries, survey results based on EIA with TES are confounded by high rates of co-infections with other soil-transmitted helminths, as antibodies to these other nematodes may cross-react to *T.canis* antigens [29],[38]. In an effort to increase both the sensitivity and specificity of TES-based EIAs, some investigators have examined the advantages of measuring IgG subclass antibodies. At least one study has shown that sensitivity could be increased by measuring IgG2 subclass antibodies, presumably those that measure anti-carbohydrate antibodies against TES glycans, while specificity could be increased by measuring IgG3 or IgG4 antibodies [29],[30]. In 2000, a 30-kDa recombinant TES antigen was cloned and expressed in bacteria [39]. The recombinant protein requires solubilization in urea (which may lessen its usability in an EIA format), but is undergoing evaluation as a potentially improved diagnostic reagent [38], as are other recombinant *T. canis* antigens [40]. Ultimately, further epidemiological studies and disease burden assessments of toxocariasis would benefit from the development of an immunodiagnostic assay that is both highly sensitive and specific for (and uses) the detection of antibodies to a chemically defined recombinant *T. canis* antigen, preferably one that is soluble in aqueous solution, and would be made widely available. Production of recombinant antigens may require expression in yeast or other low-cost eukaryotic expression vectors, which are often preferable to bacteria for producing soluble recombinant nematode antigens [41],[42]. Alternatively, tests could be developed for measuring the presence of *Toxocara* antigen in the bloodstream, similar to the immunochromatographic test (ICT) developed for lymphatic filariasis [43] or tests for other helminth infections [44].

Further studies to improve diagnostic testing and expand epidemiologic surveillance should be conducted in parallel with control and prevention efforts. These include periodic deworming of dogs (especially after whelping) and hand-washing to prevent fecal oral contact [18], and case-detection and treatment with albendazole [45]. Given the high prevalence of toxocariasis in areas of poor urban and rural hygiene [16],[21], improved sanitation and access to clean water may also have important roles. As a potential explanation for the high rates of asthma and developmental delays among disadvantaged children in poor urban and rural areas, there is an urgent need to fully explore the contribution of toxocariasis to these conditions, which in turn will require increased advocacy and resource mobilization. Recognition of toxocariasis as a common parasitic disease in the US and possibly an even greater health problem in developing countries is a first important step to national and international efforts to combat this neglected infection of poverty.
